# Early intubation and decreased in-hospital mortality in patients with coronavirus disease 2019

**DOI:** 10.1186/s13054-022-03995-1

**Published:** 2022-05-06

**Authors:** Ryo Yamamoto, Daiki Kaito, Koichiro Homma, Akira Endo, Takashi Tagami, Morio Suzuki, Naoyuki Umetani, Masayuki Yagi, Eisaku Nashiki, Tomohiro Suhara, Hiromasa Nagata, Hiroki Kabata, Koichi Fukunaga, Kazuma Yamakawa, Mineji Hayakawa, Takayuki Ogura, Atsushi Hirayama, Hideo Yasunaga, Junichi Sasaki, the J-RECOVER study group

**Affiliations:** 1 Department of Emergency and Critical Care Medicine, Keio University School of Medicine, 35 Shinanomachi, Shinjuku, Tokyo, 160-8582 Japan; 2 Trauma and Acute Critical Care Center, Tokyo Medical and Dental University Hospital, Tokyo, Japan; 3 Department of Emergency and Critical Care Medicine, Nippon Medical School Musashikosugi Hospital, Kawasaki, Kanagawa Japan; 4 Department of Clinical Epidemiology and Health Economics, School of Public Health, The University of Tokyo, Tokyo, Japan; 5 Department of Emergency and Critical Care Medicine, Kawakita General Hospital, Tokyo, Japan; 6 Emergency Medicine and Acute Care Surgery, Matsudo City General Hospital, Chiba, Japan; 7 Department of Emergency and Critical Care Medicine, Yokohama City Minato Red Cross Hospital, Yokohama, Kanagawa Japan; 8 Department of Anesthesiology, Keio University School of Medicine, Tokyo, Japan; 9 Division of Pulmonary Medicine, Department of Medicine, Keio University School of Medicine, Tokyo, Japan; 10 Department of Emergency Medicine, Osaka Medical and Pharmaceutical University, Osaka, Japan; 11 Department of Emergency Medicine, Hokkaido University Hospital, Sapporo, Hokkaido Japan; 12 Department of Emergency Medicine and Critical Care Medicine, Tochigi Prefectural Emergency and Critical Care Centre, Imperial Foundation Saiseikai Utsunomiya Hospital, Tochigi, Japan; 13 Public Health, Department of Social Medicine, Graduate School of Medicine, Osaka University, Osaka, Japan

**Keywords:** Coronavirus infection, Respiratory failure, Oxygen, Timing of intubation, Pulmonary function, Critical care

## Abstract

**Background:**

Some academic organizations recommended that physicians intubate patients with COVID-19 with a relatively lower threshold of oxygen usage particularly in the early phase of pandemic. We aimed to elucidate whether early intubation is associated with decreased in-hospital mortality among patients with novel coronavirus disease 2019 (COVID-19) who required intubation.

**Methods:**

A multicenter, retrospective, observational study was conducted at 66 hospitals in Japan where patients with moderate-to-severe COVID-19 were treated between January and September 2020. Patients who were diagnosed as COVID-19 with a positive reverse-transcription polymerase chain reaction test and intubated during admission were included. Early intubation was defined as intubation conducted in the setting of ≤ 6 L/min of oxygen usage. In-hospital mortality was compared between patients with early and non-early intubation. Inverse probability weighting analyses with propensity scores were performed to adjust patient demographics, comorbidities, hemodynamic status on admission and time at intubation, medications before intubation, severity of COVID-19, and institution characteristics. Subgroup analyses were conducted on the basis of age, severity of hypoxemia at intubation, and days from admission to intubation.

**Results:**

Among 412 patients eligible for the study, 110 underwent early intubation. In-hospital mortality was lower in patients with early intubation than those with non-early intubation (18 [16.4%] vs. 88 [29.1%]; odds ratio, 0.48 [95% confidence interval 0.27–0.84]; *p* = 0.009, and adjusted odds ratio, 0.28 [95% confidence interval 0.19–0.42]; *p* < 0.001). The beneficial effects of early intubation were observed regardless of age and severity of hypoxemia at time of intubation; however, early intubation was associated with lower in-hospital mortality only among patients who were intubated later than 2 days after admission.

**Conclusions:**

Early intubation in the setting of ≤ 6 L/min of oxygen usage was associated with decreased in-hospital mortality among patients with COVID-19 who required intubation.

*Trial Registration* None.

**Supplementary Information:**

The online version contains supplementary material available at 10.1186/s13054-022-03995-1.

## Background

Coronavirus disease 2019 (COVID-19) causes respiratory failure and often requires mechanical ventilation (MV) [1, 2]. Although several medications prevent disease progression and improve clinical outcomes [3–7], many patients still die following long-term MV management. Rapid deterioration of oxygenation is also a particular feature of COVID-19, impeding physicians from determining the optimal timing of intubation [8, 9].

The idea of early intubation with relatively preserved lung function arose based on early data, where the initiation of MV after developing severe acute respiratory distress syndrome (ARDS) had devastating consequences in patients with COVID-19 [10, 11]. Avoiding self-induced lung injury due to spontaneous breathing is another pathophysiological benefit of early intubation [12], although obvious favorable outcomes following such a strategy have not been validated [13]. Notably, some academic organizations recommended that physicians intubate patients with COVID-19 with a relatively lower threshold of oxygen usage, such as 6–8 L/min, without any scientific data [14, 15].

Given the potential benefit of early intubation, several studies compared different intubation times for respiratory failure due to COVID-19 and identified increased mortality and prolonged MV use in patients who were intubated in a later phase [16, 17]. However, most studies defined early intubation using days from admission to intubation, rather than the degree of preserved pulmonary function at the time of intubation; therefore, immortal time bias is a concern [18]. Moreover, the lack of patient characteristics at the time of intubation disturbs data interpretation, and it remains unclear whether the timing of initiation of MV simply reflects COVID-19 severity.

Therefore, we examined patients with COVID-19 who required intubation using a multicenter database to elucidate the clinical benefit of early intubation, which was defined as intubation for patients with a limited amount of oxygen usage. We hypothesized that early intubation is associated with decreased in-hospital mortality among patients with COVID-19.

## Methods

### Study design and setting

A retrospective, multicenter, observational study was conducted by the J-RECOVER study group, which was established in 2020 to investigate multiple clinical issues related to COVID-19, using data between January and September 2020 [19]. Sixty-six hospitals, where patients with moderate-to-severe COVID-19 were treated, participated in the study. Before study initiation, collaborating hospitals obtained individual local institutional review board (IRB) approval for conducting research with human subjects. This study was approved by the IRB of the Keio University School of Medicine (application number: 20200317) for conducting research with humans. The requirement for informed consent was waived because of the anonymous nature of the data used.

In Japan, after sporadic COVID-19 cases were noted in January 2020, there were two surges of newly diagnosed COVID-19 cases during the study period. During those surges, several academic organizations were concerned of nosocomial infection among healthcare providers during the invasive respiratory care of patients with COVID-19, and they recommended avoiding noninvasive positive-pressure ventilation (NIPPV) and high-flow nasal cannula (HFNC) for patients with COVID-19. Additionally, physicians at some institutions preferred to intubate patients with COVID-19 with lower thresholds of oxygen usage, such as 6–8 L/min.

### Study population

We included patients who met the following three inclusion criteria: (1) diagnosis of COVID-19 with a positive reverse-transcription polymerase chain reaction (RT-PCR) result for severe acute respiratory syndrome coronavirus-2 (SARS-CoV-2), (2) at least 18 years of age, and (3) intubated during admission. Patients were excluded if they were transferred from another health care facility after intubation or they were re-admitted for recurrent COVID-19 symptoms.

### Data collection and definitions

Participating hospitals obtained data from medical charts and the Japanese Diagnosis Procedure Combination (DPC) records at each hospital [20]. DPC is used for administrative claims and, therefore, includes demographic data; diagnosis at admission, comorbidities, and post-admission complications that are coded with the International Classification of Diseases, 10th Revision; chronic cardiopulmonary status, including Hugh–Jones and New York Heart Association (NYHA) functional classifications; treatments provided during hospitalization, including medications, blood products, surgery, and interventional procedures, along with dose and date; and discharge abstract data. Data are recorded using a uniform data submission format across the country, and physicians at each institution are mandated to confirm that data are correctly submitted with reference to medical charts. As DPC is a record for inpatients, data after hospital discharge are not available.

Data were also obtained from medical charts that included the following: the date of onset of COVID-19 symptoms, positive RT-PCR test, and admission; type of initial symptom; vital signs, hemodynamic score of Sequential Organ Failure Assessment (SOFA), arterial blood gas assay (i.e., pH, partial pressure of oxygen and carbon dioxide [PaO_2_ and PaCO_2_, respectively]), and lactate value on hospital admission; intubation data, including the amount of oxygen administered immediately before intubation, Glasgow Coma Scale (GCS) and hemodynamic score of SOFA before intubation, arterial blood gas assay before and after intubation, and initial setting of MV; days of MV and NIPPV as well as usage of HFNC; prone ventilation, extracorporeal membrane oxygenation (ECMO), and reintubation; and laboratory data measured during admission. Additionally, data regarding particular medications, including but not limited to remdesivir, complications, and cause of death were obtained from medical charts to complement the DPC data.

Early intubation was defined as intubation that was conducted when the amount of oxygen administered immediately before intubation was ≤ 6 L/min, whereas non-early intubation was defined when the amount of oxygen was > 6 L/min or when HFNC or NIPPV was used before intubation. The frequency of early intubation at each institution was calculated, and participating hospitals were categorized into the following three different frequencies of early intubation, using cutoff values that trisect the number of patients as equally as possible: low (< 20% of patients with COVID-19 underwent early intubation), moderate (20–40%), and high (≥ 40%). Blood test on hospital admission was defined as the earliest data within 7 days after admission, whereas pre-intubation blood test was defined as the data on the day of intubation.

### Outcome measures

The primary outcome was in-hospital mortality. Secondary outcomes included hospital- and ventilator-free days to day 30 after intubation, requirement of prone ventilation and ECMO, and incidence of re-intubation. Nosocomial infection of COVID-19 due to HFNC or NIPPV usage at each institution was also included in secondary outcomes.

### Statistical analysis

Patient data were classified as early intubation and non-early intubation groups based on the timing of intubation, and unadjusted analysis was performed on the primary outcome with the Chi-square test.

Inverse probability weighting (IPW) analyses with propensity scores were performed to adjust patient background between the two groups and compare the primary and secondary outcomes [21, 22]. The propensity score was developed using a logistic regression model to estimate the probability of being assigned to the early intubation group. Relevant covariates were selected from known or possible predictors for early intubation with relatively preserved pulmonary function in patients with COVID-19 [2–5, 23, 24] and included age, sex, comorbidities (Charlson index), chronic cardiopulmonary status (Hugh–Jones and NYHA functional classifications), clinical status on admission (GCS, hemodynamic score of SOFA, and oxygen requirement), days from the onset of symptoms to intubation, pre-intubation hemodynamic score of SOFA, arterial blood gas assay before intubation, and medications for COVID-19 (corticosteroid, remdesivir, and tocilizumab) before intubation. To adjust institutional characteristics, early intubation frequency was also included in the model; patients with missing covariates were excluded. Discrimination and calibration precision of the propensity score was analyzed using the c-statistic and the Hosmer–Lemeshow goodness-of-fit test, respectively [21]. IPW analyses were then performed as adjusted analyses where the primary outcome was compared with the Chi-square test [22]. Secondary outcomes were evaluated with odds ratios (ORs) or median differences using the Hodges–Lehmann estimator.

Three sensitivity analyses were conducted to examine the robustness of primary results. We performed an instrumental variable (IV) analysis to simulate the random assignment of patients to early intubation to account for unmeasured confounding [25]. The frequency of early intubation (early intubation rate) at each institution was used as an IV to examine the relationship between early intubation and in-hospital mortality. Moreover, multivariate logistic regression analysis with the forward stepwise method was performed to confirm that the results were not dependent on the propensity score calculation. IPW with restriction was also conducted to avoid extreme weights, where patients with a propensity score > 0.95 and < 0.05 were excluded [22].

Subgroup analyses analyzed the association between early intubation, clinical characteristics, and in-hospital mortality. IPW analyses were repeated in patient subgroups determined by age (< 65 vs. ≥ 65 years), severity of hypoxemia before intubation (< 60 vs. ≥ 60 mm Hg of PaO_2_), inadequate resuscitation before intubation (> 2 vs. ≤ 2 mmol/L of arterial lactate), and days from admission to intubation (≤ 2 vs. ≥ 3 days).

Descriptive statistics are presented as the median (interquartile range [IQR]) or a number (percentage). Results are shown using standardized difference and 95% confidence interval (CI), and a standardized difference < 0.1 was considered non-significant. The hypothesis was tested on the primary outcome in which a two-sided α threshold of 0.05 was considered statistically significant. Secondary outcomes were compared with Chi-square test or nonparametric median test as appropriate. All statistical analyses were conducted using IBM SPSS Statistics for Windows version 27.0 (IBM Corp., Armonk, NY), R Version 4.0.2 (R Foundation for Statistical Computing, Vienna, Austria), and Microsoft Excel (Microsoft Corp., Redmond, WA).

## Results

### Patient characteristics

**Fig. 1:**
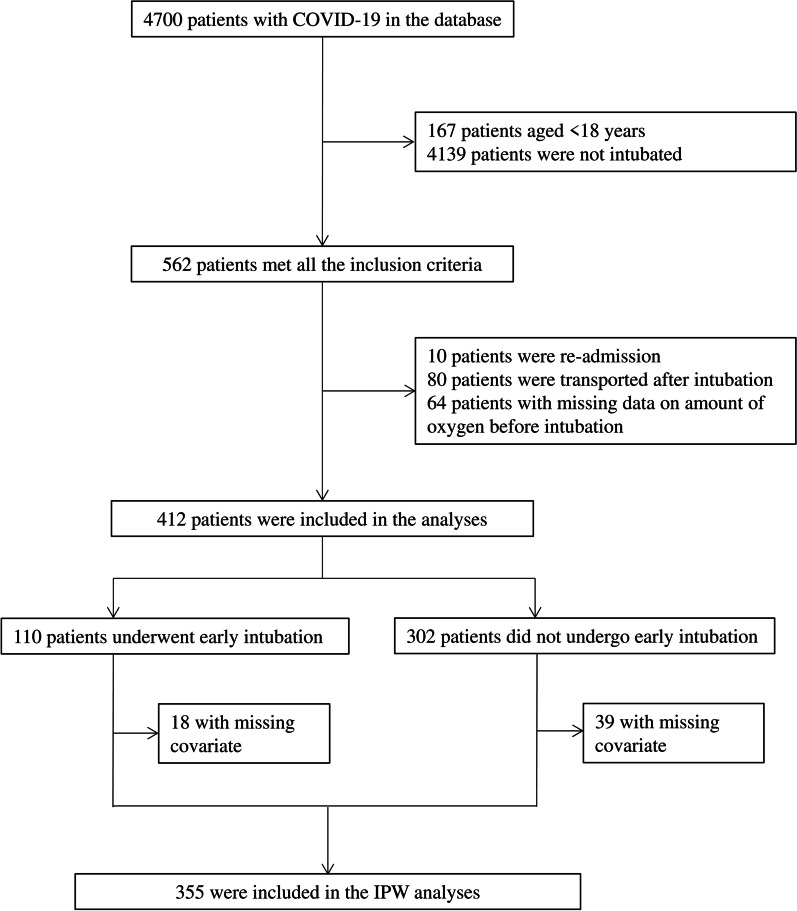
Patient flow diagram. Among 4700 patients with coronavirus disease (COVID-19) in the J-RECOVER database, 412 adult patients were intubated after hospital admission and were therefore eligible for this study. A total of 110 (26.7%) patients underwent early intubation for whom ≤ 6 L/min of oxygen was administered immediately before intubation. Eighteen patients in the early intubation group and 39 patients in the non-early intubation group were excluded from inverse probability weighting (IPW) analyses due to missing covariates for propensity score calculation; hence, IPW analyses were performed for 355 patients

Among 4700 patients with COVID-19 in the J-RECOVER database, 412 adult patients were intubated after hospital admission and were therefore eligible for this study (Fig. 1). A total of 110 (26.7%) patients underwent early intubation for whom ≤ 6 L/min of oxygen was administered immediately before intubation.

Patient characteristics are summarized in Table 1. Patients who underwent early intubation had a higher Charlson index and higher C-reactive protein and D-dimer blood levels on the day of intubation than those who did not undergo early intubation. Additionally, more patients with early intubation had a severe chronic cardiopulmonary status (Hugh–Jones classification > III and NYHA functional classification > II), ≥ 2 mmol/L of lactate at intubation, and received remdesivir and tocilizumab before intubation compared with those without early intubation. Conversely, fewer patients in the early intubation group required ≥ 4 L/min of oxygen on hospital admission. The median number of days from the onset of symptoms to intubation was 8 days in both groups, and PaO_2_ before intubation was comparable between the two groups.

**Table 1:** Characteristics of COVID-19 patients with intubation

	Before IPW			After IPW		
	Early intubation	Non-early intubation	Standardized difference	Early intubation	Non-early intubation	Standardized difference
Cases, *n*	110	302				
Age, years, median (IQR)	67 (54–74)	67 (57–76)	0.082	67 (55–71)	67 (56–76)	0.094
Sex, male, *n* (%)	79 (71.8%)	247 (81.8%)	0.238	313 (83.7%)	284 (80.7%)	0.079
Comorbidity, Charlson index, median (IQR)	0 (0–1)	0 (0–1)	0.122	0 (0–1)	0 (0–1)	0.095
Comorbidity, chronic lung disease, *n* (%)	1 (0.9%)	0 (0.0%)	0.136	0 (0.0%)	0 (0.0%)	0.000
Comorbidity, diabetes, *n* (%)	31 (28.2%)	76 (25.2%)	0.068	113 (30.2%)	98 (27.8%)	0.052
Chronic cardiopulmonary status						
Hugh–Jones classification, > III, *n* (%)	20 (18.2%)	39 (12.9%)	0.146	63 (16.8%)	53 (15.1%)	0.049
NYHA functional classification, > II, *n* (%)	1 (0.9%)	0 (0.0%)	0.136	1 (0.3%)	0 (0.0%)	0.073
Status on hospital arrival						
GCS, median (IQR)	15 (15–15)	15 (15–15)	0.098	15 (15–15)	15 (15–15)	0.000
Respiratory rate, /min, median (IQR)	22 (18–27)	24 (20–28)	0.106	24 (20–28)	24 (20–28)	0.027
Oxygen requirement, ≥ 4 L/min, *n* (%)	50 (58.1%)	171 (70.4%)	0.259	242 (73.8%)	208 (69.8%)	0.089
SOFA, hemodynamic score, median (IQR)^a^	0 (0–0)	0 (0–0)	0.219	0 (0–0)	0 (0–0)	0.000
SOFA on ICU admission, total score, median (IQR)	6 (4–8)	6 (4–9)	0.203	5 (3–6)	5 (4–7)	0.000
Status at intubation						
Days from onset of symptoms, median (IQR)	8 (5–10)	8 (6–10)	0.101	8 (6–10)	8 (6–10)	0.016
PaO_2_, mm Hg, median (IQR)	70 (59–81)	69 (58–87)	0.046	67 (57–83)	71 (59–86)	0.047
SOFA, hemodynamic score, median (IQR)^a^	0 (0–0)	0 (0–0)	0.206	0 (0–0)	0 (0–0)	0.000
Lactate, ≥ 2 mmol/L, *n* (%)	6 (37.5%)	5 (15.6%)	0.511	9 (2.4%)	6 (1.7%)	0.050
Blood test at intubation, median (IQR)						
WBC, 10^3^/μL	6.8 (4.7–8.5)	6.8 (5.2–9.5)	0.048	8.0 (5.0–8.0)	6.7 (5.5–8.5)	0.056
CRP, mg/dL	11 (5–15)	10 (7–14)	0.150	14 (7–14)	11 (7–17)	0.048
D-dimer, μg/dL	2.0 (1.1–4.7)	1.8 (0.8–2.9)	0.387	2.1 (1.5–2.1)	1.7 (0.9–2.7)	0.074
Medications, *n* (%)						
Remdesivir	37 (33.6%)	64 (21.2%)	0.282	99 (26.5%)	83 (23.6%)	0.067
Tocilizumab	9 (8.2%)	7 (2.3%)	0.265	12 (3.2%)	12 (3.4%)	0.011
Dexamethasone^b^	28 (25.5%)	78 (25.8%)	0.009	86 (23.0%)	94 (26.7%)	0.086
Respiratory support before intubation, *n* (%)						
HFNC	0 (0.0%)	25 (8.3%)	0.425			
NIPPV	0 (0.0%)	1 (0.3%)	0.082			
Frequency of early intubation, *n* (%)						
Low	6 (5.5%)	132 (43.7%)	0.992	149 (39.8%)	127 (36.1%)	0.078
Moderate	43 (39.1%)	115 (38.1%)	0.021	121 (32.4%)	120 (34.1%)	0.037
High	61 (55.5%)	55 (18.2%)	0.837	105 (28.1%)	106 (30.1%)	0.045
Days from arrival to intubation, days, median (IQR)	0 (0–3)	0 (0–2)		0 (0–1)	0 (0–2)	

*COVID-19* coronavirus disease 2019, *IPW* inverse probability weighting, *IQR* interquartile range, *NYHA* New York Heart Association, *GCS* Glasgow Coma Scale, *SOFA* Sequential Organ Failure Assessment, *ICU* intensive care unit, *PaO*_*2*_ partial pressure of oxygen, *WBC* white blood cell count, *CRP* C-reactive protein, *HFNC* high-flow nasal cannula, *NIPPV* noninvasive positive-pressure ventilation

^a^The hemodynamic score of SOFA is on a scale of 0 to 4, where 0 indicates ≥ 70 mm Hg of mean arterial pressure

^b^Other corticosteroids equivalent to 6 mg of dexamethasone (or at a least half dose of it) are included

A propensity model to predict the assignment of patients to early intubation was developed, and the discrimination and calibration were calculated, with a c-statistic of 0.821 (0.773–0.869) and Hosmer–Lemeshow goodness of fit of *p* = 0.800, respectively. Eighteen patients in the early intubation group and 39 patients in the non-early intubation group were excluded from IPW analyses due to missing covariates for propensity score calculation; hence, IPW analyses were performed for 355 patients (Fig. 1). The characteristics of patients after IPW are summarized with standardized differences in Table 1, where all covariates were successfully adjusted.

Post-intubation characteristics (Table 2) showed that patients with early intubation have a higher PaO_2_/fraction of inspired oxygen (FiO_2_) (P/F) ratio and lower PaCO_2_ than those without early intubation. In addition, fewer patients with early intubation had > 2 mmol/L of lactate after intubation. Furthermore, although the tidal volume of MV was similarly set in both groups, the peak and mean inspiratory pressures were lower among patients with early intubation.

**Table 2:** Characteristics of COVID-19 patients after intubation

	Early intubation	Non-early intubation	Standardized difference
Arterial blood gas assay			
P/F ratio, median (IQR)	195 (167–299)	159 (132–211)	0.654
PaO_2_, mm Hg, median (IQR)	120 (94–156)	96 (77–137)	0.233
pH, median (IQR)	7.37 (7.33–7.41)	7.36 (7.31–7.41)	0.000
PaCO_2_, mm Hg, median (IQR)	40 (38–42)	43 (37–48)	0.483
Lactate, ≥ 2 mmol/L, *n* (%)	17 (4.6%)	24 (7.1%)	0.108
MV setting, median (IQR)			
FiO_2_	0.5 (0.5–0.7)	0.6 (0.5–1.0)	0.509
Tidal volume, mL	450 (400–480)	430 (380–500)	0.012
Respiratory rate, breaths/min	18 (15–20)	16 (15–20)	0.196
MV measurements, median (IQR)			
Peak inspiratory pressure, cmH_2_O	23 (20–24)	24 (21–27)	0.437
Mean inspiratory pressure, cmH_2_O	14 (11–15)	14 (12–16)	0.130

The numbers in the table were adjusted by weighing with propensity scores

*COVID-19* coronavirus disease 2019, *IQR* interquartile range, *P/F* PaO_2_/FiO_2_, *PaO*_*2*_ partial pressure of oxygen, *PaCO*_*2*_ partial pressure of carbon dioxide, *FiO*_*2*_ fraction of inspired oxygen, *MV* mechanical ventilation

### In-hospital mortality and secondary outcomes

In-hospital mortality was significantly lower in patients who underwent early intubation than those who did not undergo early intubation in unadjusted analysis (18 [16.4%] vs. 87 [28.8%]; OR, 0.48 [95% CI 0.28–0.85]; *p* = 0.010; Table 3). Moreover, adjusted analysis identified similar results (9.9% vs. 27.6%; OR, 0.29 [95% CI 0.19–0.44]; *p* < 0.001; Table 3).

**Table 3:** Early intubation and clinical outcomes

	Early intubation	Non-early intubation	*p* value	OR (95% CI)	Difference in median (95% CI)
In-hospital mortality					
Unadjusted, *n*/total (%)	18/110 (16.4%)	87/302 (28.8%)	0.010	0.48 (0.28–0.85)	
IPW analysis, % (95% CI)	9.9% (6.9–12.9%)	27.6% (22.9–32.2%)	< 0.001	0.29 (0.19–0.44)	
Hospital-free days to day 30 after intubation, days, median (IQR)	23 (16–27)	14 (0–25)	< 0.001		3 (1–5)
Ventilator-free days to day 30 after intubation, days, median (IQR)	5 (0–14)	1 (0–15)	0.010		0 (0–2)
Requirement of prone ventilation, % (95% CI)	28.8% (23.8–33.8%)	33.4% (28.3–38.6%)	0.206	0.81 (0.58–1.13)	
Requirement of ECMO, % (95% CI)	1.6% (0.3–2.9%)	14.4% (10.8–18.1%)	< 0.001	0.10 (0.04–0.23)	
Re-intubation, % (95% CI)	10.6% (7.3–14.0%)	7.7% (4.3–11.2%)	0.244	1.42 (0.78–2.58)	
Nosocomial infection of COVID-19 due to HFNC/NIPPV, per institution, *n*/total (%)					
Pre-intubation usage		0/16 (0.0%)			
Post-intubation usage	0/2 (0.0%)	0/5 (0.0%)			

*OR* odds ratio, *CI* confidence interval, *IPW* inverse probability weighting, *IQR* interquartile range, *HFNC* high-flow nasal cannula, *NIPPV* noninvasive positive-pressure ventilation, *MV* mechanical ventilation, *ECMO* extracorporeal membrane oxygenation, *COVID-19* coronavirus disease 2019

Early intubation was also associated with less frequent ECMO usage (1.6% vs. 14.4%; OR, 0.10 [95% CI 0.04–0.23]; Table 3), whereas the frequency of prone ventilation was similar regardless of the timing of intubation. Furthermore, hospital- and ventilator-free days to day 30 after intubation were related to early intubation, while there were no differences in the incidence of re-intubation. Moreover, nosocomial infection of COVID-19 due to HFNC or NIPPV usage at each institution was not identified in either group (Table 3).

Sensitivity analysis using IV identified a relationship between early intubation and decreased in-hospital mortality (OR, 0.46 [95% CI 0.23–0.90]; Additional file 1: Table S1), where the early intubation rate at each institution was strongly associated with early intubation, but not with in-hospital mortality. Multivariate logistic regression and IPW with restriction similarly revealed that early intubation was associated with decreased in-hospital mortality (OR, 0.38 [95% CI 0.24–0.60] and OR, 0.49 [95% CI 0.32–0.77], respectively; Additional file 1: Table S1).

### Subgroup analysis

In subgroup analyses (Table 4), a relationship between reduced in-hospital mortality and early intubation was observed in several subgroups, namely elderly (≥ 65 years) and non-elderly adults (< 65 years) and severe hypoxemia before intubation (PaO_2_ < 60 mm Hg) and non-severe hypoxemia (PaO_2_ ≥ 60 mm Hg).

**Table 4:** In-hospital mortality in subgroup analyses

	Early intubation	Non-early intubation	OR	95% CI
Age				
< 65 years	1.7% (0.0–3.7%)	11.1% (6.1–16.1%)	0.14	0.04–0.50
≥ 65 years	16.7% (11.6–21.9%)	40.0% (33.2–46.8%)	0.30	0.19–0.48
Severity of hypoxemia before intubation				
PaO_2_ < 60 mm Hg	3.4% (0.1–6.7%)	39.7% (27.6–51.8%)	0.05	0.02–0.16
PaO_2_ ≥ 60 mm Hg	12.7% (7.6–17.7%)	26.7% (19.9–33.4%)	0.40	0.23–0.71
Inadequate resuscitation before intubation				
Lactate ≤ 2 mmol/L	6.8% (3.7–2.9%)	28.7% (22.0–35.5%)	0.47	0.16–1.37
Lactate > 2 mmol/L	20.0% (5.7–34.3%)	34.7% (21.4–48.0%)	0.18	0.10–0.33
Days from admission to intubation				
≤ 2 days	9.5% (6.2–12.7%)	27.9% (22.6–33.2%)	0.27	0.17–0.43
≥ 3 days	12.3% (3.8–20.8%)	26.0% (16.2–35.8%)	0.40	0.16–1.02

Inverse probability weighting analyses were performed in each subgroup and presented as % (95% CI)

*OR* odds ratio, *CI* confidence interval, *PaO*_*2*_ partial pressure of oxygen

Conversely, patients with ≤ 2 mmol/L of pre-intubation lactate have comparable mortality regardless of intubation timing, whereas those patients with > 2 mmol/L of lactate have significantly lower in-hospital mortality when they underwent early intubation. Moreover, when patients were intubated within 2 days after hospital admission, in-hospital mortality was similar between groups; however, when they were intubated later than 2 days, early intubation was associated with decreased in-hospital mortality.

## Discussion

In this study, early intubation in a setting of ≤ 6 L/min of oxygen usage was associated with decreased in-hospital mortality among patients with COVID-19 who underwent intubation. Importantly, this relationship remained after adjusting for patient background and disease severity at the time of intubation; multiple sensitivity analyses also confirmed the robustness of our results.

Several pathophysiological mechanisms underlying the relationship between early intubation and reduced in-hospital mortality can be considered. First, the early initiation of positive-pressure ventilation prevents alveolar injury caused by the negative pressure of spontaneous breathing. Among patients with hypoxemic respiratory failure, including ARDS, patient self-induced lung injury happens by high transpulmonary pressure due to spontaneous inspiratory drive [12, 26, 27]. An animal study also reported that spontaneous breathing caused overstretch of the dependent lung, followed by alveolar injuries [28, 29]. Notably, patients with non-early intubation in this study had more severe lung injury (i.e., lower post-intubation PaO_2_, higher PaCO_2_, and higher peak and mean inspiratory pressures), whereas pre-intubation PaO_2_ was comparable between early and non-early intubation groups. Considering that patients with COVD-19 who require MV frequently present with vigorous spontaneous breathing [2, 30], early lung-protective ventilation may further mitigate alveolar injury in this study.

Second, oxygenation deterioration during intubation affects the survival of patients with COVID-19. Prolonged hypoxemia after intubation was reported in patients with severe COVID-19 [31], and the post-intubation P/F ratio was lower in patients with non-early intubation in this study, suggesting that aerated lung tissue was considerably reduced in such a population. Further, insufficient preoxygenation during intubation contributes to unfavorable outcomes in patients with non-early intubation [32].

Third, given that fewer patients with early intubation had > 2 mmol/L of lactate after intubation than those with non-early intubation, early intubation may minimize the hemodynamic instability of patients with severe COVID-19. As lung injury progression increases the positive pressure needed to open the collapsed lung [27, 28], MV usage for relatively preserved lung tissue among patients with early intubation may have less disturbance on the hemodynamic stability. In addition, this speculation would be reflected in the results of subgroup analyses, in which only patients with > 2 mmol/L of pre-intubation lactate benefited from early intubation. Hemodynamic disturbance during non-early intubation might have been manifested only among patients with preexisting hemodynamic instability.

Patient who underwent early intubation had higher number of hospital- and ventilator-free days, while requirement of prone ventilation and incidence of re-intubation did not differ between early and non-early intubation groups. However, these results need to be interpreted with caution because the study size is limited. It should be emphasized that nosocomial infection of COVID-19 from HFNC or NIPPV usage was identified in this study population.

Most previous studies on early versus delayed intubation defined “early” as within 24–48 h after hospital admission and reported potential benefits of an early intubation strategy [16, 33, 34]. However, these retrospective studies introduced considerable discussion because the definition of early intubation by the timing during hospital stay may reflect the rapidness of disease exaggeration, rather than the treatment strategy. The present study defined patients based on oxygen usage at the time of intubation, and reduced in-hospital mortality was found in patients with early intubation after adjusting for disease severity, degree of inflammation, and cardiopulmonary status at intubation. Furthermore, this benefit was observed only when patients were intubated later than 2 days after admission, suggesting that patients who needed intubation within 48 h after admission have unique features. Considering the results in this study, early intubation should be further investigated as a useful strategy in patients with COVID-19.

Our results must be interpreted within the context of the study design. We retrospectively retrieved data, which do not record the indications of early intubation instead of non-early intubation. Therefore, our results may differ if the decision of early intubation with relatively preserved pulmonary function is dependent on unrecorded strong prognostic factors, such as the quality of critical care. However, it should be emphasized that IV analysis was conducted as a sensitivity analysis and the association between early intubation and decreased in-hospital mortality was revealed even after unmeasured confounders were adjusted. Another limitation is that details of clinical information related to early intubation, including the degree of lung injury and transient hypoxia at the intubation, were not available. Although the post-intubation P/F ratio and inspiratory pressures were recorded, the potential consequences of such parameters following early and non-early intubation cannot be evaluated on the basis of objective data. In addition, while work of breathing may influence decision for early intubation, we were not able to incorporate this into our model due to the absence of respiratory rate or respiratory rate oxygenation (ROX) index in our database. Moreover, we investigated only patients who required intubation. Considering that some patients who did not undergo early intubation could recover from respiratory failure and avoid intubation, our result may overestimate the beneficial effect of early intubation. Finally, as this study included patients in early months of the pandemic year, few patients used HFNC/NIPPV. Therefore, superiority of early intubation to HFNC/NIPPV was not examined in this study and our results do not deny the usefulness of HFNC/NIPPV for patients with COVID-19. Given that HFNC/NIPPV has been shown to effectively prevent intubation in patients with COVID-19[35, 36], as well as that early intubation strategy would deplete valuable resources including ventilator, early intubation should be carefully considered in daily practice. A properly designed study is mandated to validate that early intubation before critical exaggeration of lung function is a viable treatment option in COVID-19.

## Conclusions

We revealed that early intubation in the setting of ≤ 6 L/min of oxygen usage was associated with decreased in-hospital mortality among patients with COVID-19 who required intubation. Relatively earlier intubation before pulmonary function is devastated due to COVID-19 should be further validated in future studies.

## Supplementary Information

**Additional file 1: Table S1.** In-hospital mortality in sensitivity analyses.

## Data Availability

The data of this study are available from the J-RECOVER study group; however, restrictions apply to the availability of these data, which were used under the license for the current study and so are not publicly available. However, data are available from the authors upon reasonable request and with permission of the J-RECOVER study group.

## Abbreviations

COVID-19: Coronavirus disease 2019
MV: Mechanical ventilation
ARDS: Acute respiratory distress syndrome
IRB: Institutional review board
NIPPV: Noninvasive positive-pressure ventilation
HFNC: High-flow nasal cannula
RT-PCR: Positive reverse-transcription polymerase chain reaction
SARS-CoV-2: Severe acute respiratory syndrome coronavirus-2
DPC: Diagnosis procedure combination
NYHA: New York Heart Association
SOFA: Sequential Organ Failure Assessment
PaO_2_: Partial pressure of oxygen
PaCO_2_: Partial pressure of carbon dioxide
GCS: Glasgow Coma Scale
ECMO: Extracorporeal membrane oxygenation
IPW: Inverse probability weighting
IQR: Interquartile range
CI: Confidence interval
