# Supplementary material for: Early intubation and decreased in-hospital mortality in patients with coronavirus disease 2019
Source: Crit Care. 2022 May 6;26:124. doi: 10.1186/s13054-022-03995-1 (PMC9073819; doi:10.1186/s13054-022-03995-1)
Supplement: Supplementary file 1 — Additional file 1: Table S1. In-hospital mortality in sensitivity analyses. [file 13054_2022_3995_MOESM1_ESM.docx]

| Table S1. In-hospital Mortality in Sensitivity Analyses | | | | | |  |
| --- | --- | --- | --- | --- | --- | --- |
|  |  | Early Intubation | Non-early Intubation | OR | 95% CI |  |
| In-hospital mortality, % (95% CI) | |  |  |  |  |  |
|  | Instrument variable |  |  | 0.46 | 0.23–0.90 |  |
|  | Multivariate logistic regression*^a^* |  |  | 0.38 | 0.24–0.60 |  |
|  | IPW with restriction*^b^* | 14.2% (9.9%–18.4%) | 25.1% (19.9%–30.3%) | 0.49 | 0.32–0.77 |  |
| OR = odds ratio, CI = confidence interval, and IPW = inverse probability weighting.  *^a^*The final model included early intubation, age, Glasgow Coma Scale, and hemodynamic score of Sequential Organ Failure Assessment on hospital arrival; remdesivir usage before intubation; and days from admission to intubation, as validated mortality predictors.  *^b^*Patients with a propensity score <0.05 and >0.95 were excluded, in which about 75% of the study population was selected. | | | | | |  |
|  |  |  |  |  |  |  |
|  |  |  |  |  |  |  |
|  |  |  |  |  |  |  |
